# Chemical Partition of the Radiative Decay Rate of Luminescence of Europium Complexes

**DOI:** 10.1038/srep21204

**Published:** 2016-02-19

**Authors:** Nathalia B. D. Lima, José Diogo L. Dutra, Simone M. C. Gonçalves, Ricardo O. Freire, Alfredo M. Simas

**Affiliations:** 1 Departamento de Química Fundamental, CCEN, UFPE, 50590-470, Recife, PE, Brazil; 2 Pople Computational Chemistry Laboratory, Departamento de Química, CCET, UFS, 49100-000, São Cristóvão, SE, Brazil

## Abstract

The spontaneous emission coefficient, A_rad_, a global molecular property, is one of the most important quantities related to the luminescence of complexes of lanthanide ions. In this work, by suitable algebraic transformations of the matrices involved, we introduce a partition that allows us to compute, for the first time, the individual effects of each ligand on A_rad_, a property of the molecule as a whole. Such a chemical partition thus opens possibilities for the comprehension of the role of each of the ligands and their interactions on the luminescence of europium coordination compounds. As an example, we applied the chemical partition to the case of repeating non-ionic ligand ternary complexes of europium(III) with DBM, TTA, and BTFA, showing that it allowed us to correctly order, in an *a priori* manner, the non-obvious pair combinations of non-ionic ligands that led to mixed-ligand compounds with larger values of A_rad_.

Almost 40 years ago, systems containing lanthanide ions started attracting the interest of many research groups around the world^1^,^2^ due to their very peculiar luminescence properties. Such properties make these ions, such as trivalent europium, essential for the development of photo and electroluminescent devices^3^,^4^. Since intra-ion f-f transitions are Laporte forbidden, the light absorption capability of the lanthanide ions is poor and therefore luminescence cannot be generated from direct excitation. However, when the lanthanide ion is surrounded by coordinating ligands who can effectively absorb radiation and subsequently transfer the energy, in an intramolecular manner, to the coordinated lanthanide ion – a process known as the antenna effect^5^ – then something entirely different happens. Asymmetries in the ligand field, asymmetries due to different spatial arrangement of the ligands around the lanthanide ion, asymmetries due to mixed ligands coordination^6^, asymmetries due to thermal vibrations, etc, all make the intra-ion f-f decay less forbidden, and, as a result, strong luminescence ensues. Nevertheless, this strong luminescence is still a result of an almost forbidden intra-ion f-f transition, which therefore both exhibits long lifetimes and high color purity. Indeed, the exhibited luminescence is essentially monochromatic. Moreover, since the lanthanide trication 4f orbitals belong to an inner shell which is shielded by the outer 5s^2^5p^6^ shell, this luminescence displays a high degree of insensitivity to environmental quenching, and is of value for applications in sensors^7^, displays^3^, fluoroimmunoasssays^8^, fluorescence microscopy^9^, etc.

The rapid technological development of the last decades^10^,^11^ has contributed to escalating the interest in lanthanide complexes. So much so, that, currently, there are about one thousand articles published every year on the subject^12^. However, less than 5% of the studies involving lanthanide ions make use of theoretical tools^12^. Even though, it is possible to verify an increase in the use of theoretical tools assisting the experimental studies in their quest to design increasingly more efficient luminescent systems^13^,^14^,^15^,^16^. Hence, the extant theoretical tools and techniques are slowly becoming increasingly popular, such as the Sparkle Models^17^,^18^,^19^,^20^,^21^ and RM1 for lanthanides^22^,^23^,^24^,^25^, which are fully available in the MOPAC software^26^, as well as the new lanthanide luminescence software package LUMPAC (www.lumpac.pro.br)^12^, the first and only software dedicated to the study of the luminescence properties of systems containing europium ions.

Recently, we showed that the charge factors of the simple overlap model and the polarizabilities of Judd-Ofelt theory can be uniquely modeled by perturbation theory on the semiempirical electronic wavefunction of the complex^27^. Consequently, the terms of the intensity parameters related to dynamic coupling and electric dipole mechanisms are made unique, leading to a unique set of intensity parameters per complex.

Luminescence of europium complexes is a phenomenon entirely dependent on the chemical nature of the ligands and on the fine details of their geometric arrangements, as they are coordinated to the central metal ion. Nevertheless, luminescence is still a property of the complex as a whole. Hence, the emission spectrum of the complex conceals a profusion of ligand contributions to the luminescence phenomenon. For example, by examining the luminescence properties, it is not always entirely clear which ligand should replace another, in a given compound, in order to design a novel and more luminescent complex.

In the present article, we are advancing a novel formalism for a partition of the radiative decay rate of luminescence of europium complexes into ligand contributions. Such a chemical partition scheme is shown to be general, and applicable to any europium complex. Finally, we exemplify the usage of this novel chemical partition for the choice of the best couple of non-ionic ligands for the design of mixed ligand complexes with boosted luminescence.

## Intensity parameters from experiment

Luminescence in europium complexes is a process by which ultraviolet light is converted into visible light, mostly in the red-orange region of the spectrum. The first step of this light conversion is the absorption of the ultra-violet light by high-absorbance ligands. Then, the absorbed energy is transferred to the central trivalent europium ion that, as a result, is placed in its ^5^D_0_ excited state. From that excited state, two types of processes occur: radiative decays to the low-lying ^7^F_J_ states and nonradiative decays to the ground state. This final step of the luminescence phenomenon is expressed mathematically in terms of the emission efficiency η = A_rad_/(A_rad_ + A_nrad_), where A_rad_ and A_nrad_ are, respectively, the radiative and nonradiative total decay rates.

The radiative decay rate A_rad_ is the sum of all radiative decay rates from the ^5^D_0_ excited state to each of the ^7^F_J_ states, with J = 0,1,2,3,4,5,6, whereas the non-radiative rate, A_nrad_, is actually a bundle of non-radiative processes. Since we are not interested in non-radiative decays, there is no need to identify any of their terms.

The experimental radiative decay rate, 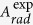, is the sum of the radiative decay rates of each of the possible transitions ^5^D_0_ → ^7^F_J_ with J ranging from 0–6:


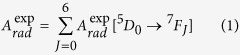


The transition ^5^D_0 _→ ^7^F_1_ is governed by a magnetic dipole mechanism and is therefore insensitive to electric dipole contributions. This transition is thus regarded as insensitive to the essentially static electric fields produced by the ligands and can be determined through the expression^28^ 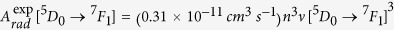, where n is the refractive index of the medium, and 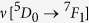 is the barycenter, the weighted mean of the frequencies in cm^−1^, corresponding the ^5^D_0_ → ^7^F_1_ transition. From this value, we can now compute the other radiative decay rates, with J from 0–6:


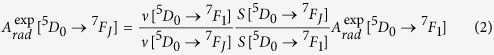


where 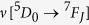 are the energies of the barycenters of the respective transitions; and S[^5^D_0_ → ^7^F_J_] are the areas under the spectra corresponding to the respective transitions. Finally, the experimental intensity parameters can be calculated from^28^:


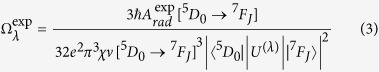


where 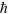 is Planck-Dirac constant, *e* is the fundamental electric charge, 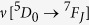 is the frequency of the transition in wavenumbers, χ is the Lorentz local-field correction term given by χ = n(n^2^ + 2)^2^/9, and 
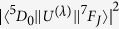
are the square reduced matrix elements whose values are 0.0032, 0.0023, and 0.0002 for λ = 2, 4, and 6, and J = λ in the case of europium^29^.

Another way of obtaining the 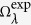 is from absorption spectra^30^. In such a procedure, the 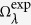
are obtained by parameterization so that the calculated oscillator strengths match the experimental ones from the absorption spectra, and the parameterization error propagates to the intensity parameters. Besides, further errors may derive from uncertainties in the choice of theoretical transitions to relate to the experimental ones. Indeed, when Judd-Ofelt parameters are estimated from absorption spectra in this manner, errors are of the order of 10–20%^30^ and that is why, in this article, we chose to evaluate them from emission spectra instead.

## Theoretical intensity parameters

The theoretical radiative decay rate for the forced electric dipole and magnetic dipole governed transitions,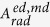, is given by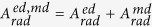, where, for europium,


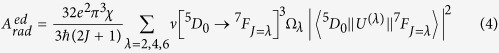


and 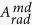 is equal to


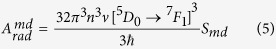


where *e* is the elementary charge; 2J + 1 is the degeneracy of the initial state, in this case ^5^D_0_, and therefore J = 0. Transitions ^5^D_0_ → ^7^F_J_ with J = 0, 3, and 5 depend on contributions from both electric and magnetic dipole mechanisms and thus are not as easy to calculate. Fortunately, their intensities are very low and, thus, they can be safely disregarded. Transition ^5^D_0_ → ^7^F_1_ is the only one which does not have an electric dipole contribution, therefore, is not sensitive to the presence of the ligands around the europium ion, and is relatively small: its magnetic dipole strength is theoretically evaluated as being *S*_*md*_ = 96 × 10^–42^ esu^2^ cm^2 ^31. Therefore, for the purpose of this work, we define 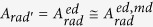, restricted to the chemically interesting transitions ^5^D_0_ → ^7^F_J_ with J = 2, 4, and 6. Their corresponding theoretical intensity parameters Ω_λ_ (λ = 2, 4, 6) emerge from the Judd-Ofelt theory^32^,^33^, depend on the coordination interaction between the lanthanide cation and the ligands, and are given by the following expression:


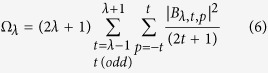


The *B*_*λ,t,p*_ terms in eq. (6) are given by the following expression:


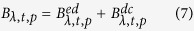


where 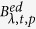
corresponds to the forced electric dipole contribution and 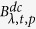 corresponds to the dynamic coupling contribution, respectively given by:


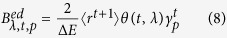



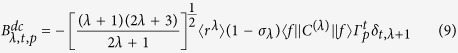


where ΔE is a constant, approximately given by the energy difference between the barycenters of the ground 4f ^N^ and first opposite parity excited state of configuration 4f ^(N−1)^5d of the europium ion; 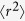 = 2.567541 × 10^−17^cm^2^ and 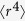 = 1.58188 × 10^−33^ cm^4^ are radial integrals, pre-defined for the europium ion^34^; θ(t,λ) are numerical factors for a given lanthanide, estimated by Malta *et al.*^35^ from Hartree-Fock calculations of the radial integrals as being: θ(1,2) = −0.17; θ(3,2) = 0.345; θ(3,4) = 0.18, θ(5,4) = −0.24, θ(5,6) = −0.24, θ(7,6) = 0.24; γ^t^_p_ are the odd-rank ligand field parameters; (1 − σ_λ)_ is a shielding factor due to the filled 5 s and 5p sub-shells of the lanthanide ion^35^, with σ_2_, σ_4_, and σ_6_ being, respectively, 0.6, 0.139, and 0.100 for Eu(III), as previously calculated by Malta and Silva^36^; 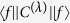
is a Racah tensor operator of rank λ whose values for λ = 2,4,6, are −1.3660, 1.128, and −1.270, respectively, for any lanthanide; 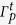 is also a sum over coordinating atoms which further reflects the chemical environment; finally, δ_t,λ+1_ is a Kronecker delta symbol. All these pre-defined parameters are taken as constants for all europium complexes.

The odd rank ligand field parameters are, in turn, given by:


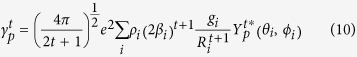


where the term 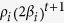, according to the Simple Overlap Model (SOM)^37^,^38^, introduces a correction to the crystal field parameters of the point charge electrostatic model (PCEM)^39^, 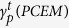, such that 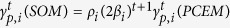, which confers a degree of covalency to the point charge model through the inclusion of parameter ρ, since PCEM only treats the metal-ligand atom bonds as a purely electrostatic phenomenon; *g*_*i*_ is the charge factor associated to the lanthanide-ligand atom bond; 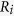 is the lanthanide-ligand atom bond distance; and 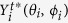 are complex conjugate spherical harmonics.

The other odd-rank parameter 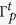, which further reflects the chemical environment is given by:


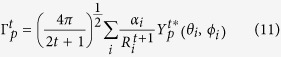


where *α*_*i*_ is the polarizability associated to the lanthanide-ligand atom bond.

In a recent article^27^, we introduced a protocol to model the charge factors g_j_ of the simple overlap model by electron densities, and the polarizabilities α_i_ of Judd-Ofelt theory by superdelocalizabilities, all obtained by perturbation theory on the semiempirical electronic wavefunction of the complex. A fitting of the theoretical intensity parameters 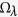 is then carried out, which reproduces the experimentally obtained 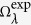 using only three adjustable constants: Q, D, and C, which must obey the acceptance criterion D/C > 1 leading to a unique adjustment. Whenever D/C ≤ 1, it was shown that the presumed geometry of the coordination polyhedron is not seemingly compatible with the experimental intensity parameters and requires improvement, either via calculation by another theoretical model, such as another Sparkle Model^17^,^18^,^19^,^20^,^21^ or RM1 model for lanthanides^22^, or via X-ray crystallographic measurements, etc. The importance of this previous work is that all derived quantities become also uniquely determined for a given complex geometry^27^, including the chemical partition that is being advanced in this article.

## Results and Discussion

### Partitioning A_rad_ into ligand terms

According to the theory, 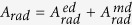. The first term, 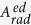, with even J, is mainly driven by electric dipole transitions. The second term, 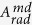, with odd J, is mainly driven by magnetic dipole transitions. Besides, recall that the strengths of the transitions ^5^D_0_ → ^7^F_J_ with J = 0, 3, and 5 are set at zero because of their low values. Likewise, the magnetic dipole driven transition ^5^D_0_ → ^7^F_1_ is not sensitive to the ligands and, as a result, is not directly relevant from a chemical point of view. Therefore, the partition will focus on the electric dipole driven transitions with J = 2, 4, 6. Accordingly, we will partition a subset of A_rad_, we call A_rad′_, defined by:


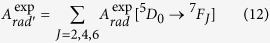


the most significant term being the decay rate of the so-called hypersensitive transition ^5^D_0_ → ^7^F_2_ , which is highly susceptible to the presence of the ligands.

Now, let us turn to compute the 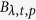 terms from eqs (8, 9, 10, 11).


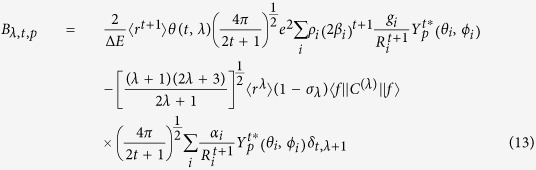


which can be rewritten as,


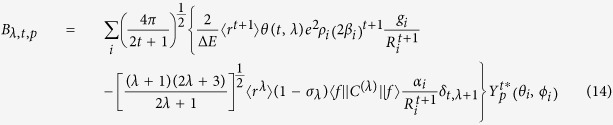


Accordingly, 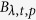 are obtained by sums over all coordinating atoms of the ligands of the product of a term, 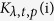, defined below, which depends only on the lanthanide ion and on the particular coordinating bond as previously described, with a complex conjugate spherical harmonic.


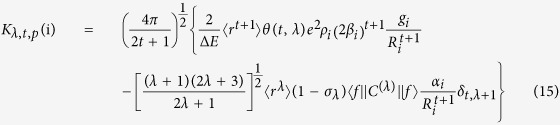


Therefore,


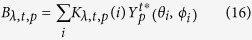


and we can define an auxiliary matrix, 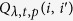, which is a function of only the coordinating atoms of the complex as


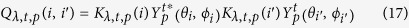


Note that all diagonal elements 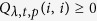. Besides, 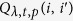 is an Hermitian matrix because 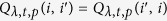
*. Therefore, its eigenvalues are all real numbers. Moreover, we will show that 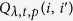 is a positive semi-definite matrix and therefore all its eigenvalues are, not only real, but also equal to or greater than zero. *Q* is said to be positive semi-definite if 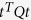 is non-negative for every non-zero column vector *t* of *n* real numbers. Here *t*^*T*^ denotes the transpose of *t*.

As such,


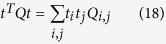


Replacing *Q* by its expression,


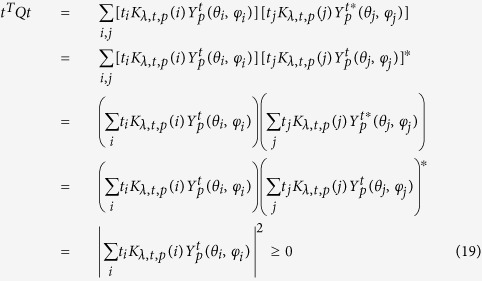


That is, 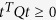. Therefore, *Q* is positive semi-definite.

The 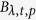 terms of eq. (16) can now be computed in terms of the directly coordinating atoms as:


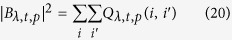


then,


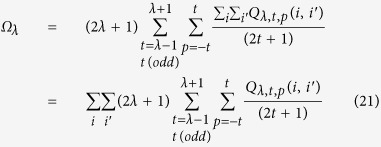


Likewise, we can define the efficacy of luminescence matrix 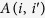 as


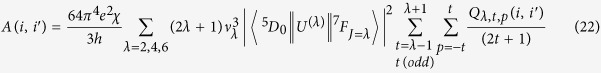


where ν_λ_ is the frequency (in cm^−1^) corresponding to the energy gap between the initial ^5^D_0_ and final ^7^F_J_ states.

Note that 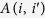 is a real symmetric positive semi-definite matrix, since 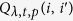 is also a real symmetric positive semi-definite matrix and the coefficients multiplying 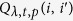 in eq. (22) are all positive. The diagonal terms 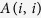 are coordinating atom contributions, and the off diagonal terms 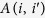 with 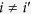, are the coordinated atom pair contributions to A_rad_[^5^D_0_ → ^7^F_J_], and, ultimately, indirect contributions to the emission efficiency. As a result,


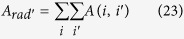


From the point of view of coordination chemistry, it is more useful to aggregate all contributions from each ligand in the complex and define the ligand contributions to the radiative decay rate by matrix 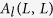, in terms of summations over the coordinating atoms k of ligand *L* as:


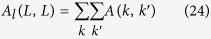


Likewise, we can define the ligand pair contribution to the radiative decay rate by matrix 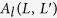, which is a measure of how well the ligands interact to enhance the radiative decay rate, in terms of summations over the coordinating atoms k and m, of ligands *L* and *L*′, respectively, as:


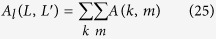


Note that the ligand-pair contributions do not contain any atomic contributions, since, by being different ligands, *L* and *L*’ do not share any directly coordinating atom.

Finally,


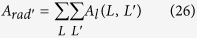


We will use the efficacies of luminescence, or simply efficacies 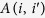 and 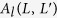 in order to interpret, from a chemical point of view, the various influences of the ligands and of their atoms, together with their pair influences, directly on 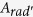. The elements of both efficacy matrices *A* and *A*_*l*_ are partial decay rates (see eqs (23) and (26)), being expressed in units of a decay rate, usually s^−1^. So, in order to obtain the total decay rate in s^−1^, just add all elements of either matrix, that is, their grand sums 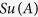 or 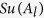 defined as 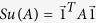 and 
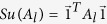
, where 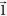
is a column vector with all elements equal to unity.

### The chemical partition of A_rad′_

The elements of the efficacy matrices *A* and *A*_*l*_, are often negative, which require an interpretation, which may sometimes be useful, but is certainly less chemically intuitive, of the partition of 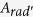. For example, if a given complex displays a very low luminescence, that is an 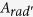 close to zero, it is possible that the contribution *A*_*l*_ from one of its ligands be +800 s^−1^ and the *A*_*l*_ of another ligand be −800 s^−1^. Such a situation in which one contribution annihilates the other, renders the role of each of the ligands on the luminescence phenomenon somewhat indiscernible, especially when they are chemically identical.

A chemically more intuitive partition would require only coordinated atom or ligand contributions, always positive, and hence, whenever 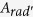 is zero, they all should be zero.

In order to define such a partition, we start by recognizing that matrix *Q* is Hermitian and positive semi-definite, and, as a consequence, matrix 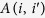 is also Hermitian and positive semi-definite. That implies that their eigenvalues are not only all real, but they are all greater than or equal to zero. Now, define the orthonormal eigenvectors of 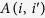 as *U*_*1*_, *U*_*2*_, …, *U*_*n*_. Let 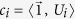, where 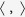 denotes an inner product. Then, 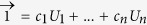. Let the eigenvalues associated to the eigenvectors *U*_*1*_ … *U*_*n*_ be, respectively, *λ*_*1*_, … *λ*_*n*_. Thus, 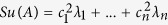. In fact,


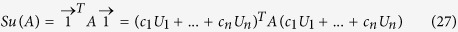



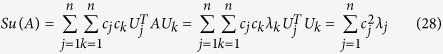


Let,


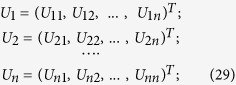


Observe that


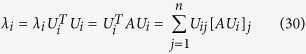


So, we define the relative contribution of the coordinated atom j to the eigenvalue λ_i_ as


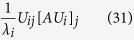


We will now obtain a nicer expression for this relative contribution. Since 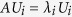, we have 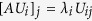, thus 
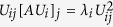
. Finally, 
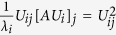
. Thus, 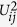
is the relative contribution of the coordinated atom *j* to eigenvalue *λ*_*i*_ because these relative contributions are non-negative and sum 1, and thus can be viewed as proportions as we intended. We can now define the vectors with the ligand contributions to the eigenvectors:


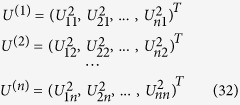


In a similar manner, we can define the absolute contribution of coordinated atom j to 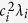 as 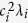
times the relative contribution of coordinated atom j to the eigenvalue λ_*i*_, i.e 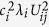. Finally, we can define the absolute contribution of coordinated atom j to Su(A), Λ_j_, as the sum of the contributions of coordinated atom j over all values 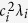, with *i* = 1…*n*. Let 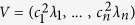. Then, the contribution of coordinated atom j to Su(A), Λ_j_, is given by


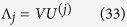


due to the definition above, 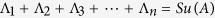, with 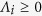.

The set of coefficients Λ_j_ thus constitutes a partition of 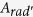 in *n* terms, all positive, each corresponding to each of the n coordinated atoms. These terms reflect how each coordinated atom contributed to make the ^5^D_0_ → ^7^F_J_ less forbidden.

As before, we can aggregate all contributions from each ligand in the complex and define the ligand contributions to 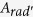 in terms of summations over the coordinating atoms *k* of ligand *L* as:


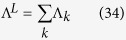


Of course,


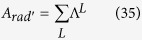


In summary, the chemical partitions we introduce in this article, 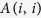, 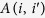, 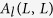, 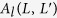, Λ_j_, and Λ^L^, are from now on available, ready to be interpreted from a myriad of chemical perspectives depending on the system of interest, subject to the creativity of the researcher.

### LUMPAC chemical partition implementation

The luminescent software package LUMPAC^12^, since 2013 freely available from http://www.lumpac.pro.br/, is the first state of the art complete software to treat europium luminescence from a theoretical point of view. Recently, the unique adjustment of theoretical intensity parameters developed by our group^27^ was implemented in LUMPAC. Now, the chemical partition being advanced in this article has also been implemented and is already available to all users of LUMPAC.

From eqs (10) and (11), clearly geometry makes a profound impact on the calculation of the theoretical intensity parameters Ω_2_, Ω_4_ and Ω_6_, and, by extension makes an impact on our partition scheme. Therefore, users must first determine the most stable geometry of the complex of interest via either RM1^22^ or any of the Sparkle Models^17^,^18^,^19^,^20^,^21^ in such a manner as to satisfy the binary outcome acceptance conditions for the unique adjustment of theoretical intensity parameters as described in the “LUMPAC implementation” section of ref. 27. Once the adjustment is considered accepted, calculation of the chemical partition follows in a seamless manner.

The partition is first computed per directly coordinated atom, and then subsequently aggregated per ligand by summing up the terms of the directly coordinated atoms of each ligand. It has been proven useful to further aggregate the terms of the ligands into terms for classes of ligands, such as the terms of all ionic ligands and those of all non-ionic ligands.

### Interpretation of the chemical partition ligand terms

Interpretation of the ligand terms requires an understanding of the fact that, according to Laporte rule, the electronic f-f transitions in lanthanide complexes should be forbidden in centrosymmetric molecules, since they conserve parity with respect to the inversion center where the metal is located. In this sense, luminescence happens because the centrosymmetry can be broken by ligands coordinating the lanthanide ion. Since luminescence happens in the europium ion, not at the ligands, the ligand terms of the 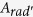 chemical partition cannot possibly be regarded as ligand contributions to 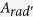, but rather as measures of the relaxation of the forbidding character of the ^5^D_0_ → ^7^F_J_ transitions (J = 2, 4, 6), conferred by each of the respective ligands to the europium ion. Note that each ligand term of the chemical partition is defined within the distinctive chemical ambiance of the particular complex, and cannot be expected to be transferable from complex to complex.

### Sensitivity of the chemical partition to complex geometry

We now turn to exemplify aspects of our partition scheme by first studying three specific complexes of the general formula Eu(β-diketonate)_3_(TPPO)_2_, where TPPO is the non-ionic ligand triphenylphosphine oxide and β-diketonate stands for one of the ionic ligands TTA, 1-(2-thenoyl),3,3,3-trifluoroacetone, BTFA, 4,4,4-trifluoro-1-phenyl-2,4-butanedione, or DBM, 1,3-diphenylpropane-1,3-dione.

A complex of this general formula Eu(β-diketonate)_3_(TPPO)_2_ may display two possible ligand arrangements: both TPPOs are either adjacent or opposite to each other. All structural data have been computed by either RM1^22^, or, in a single case, by the Sparkle/PM3^18^ method. The choice of method followed the QDC acceptance criterion^27^ defined in a previous article on the unique adjustment of theoretical intensity parameters^27^. We will now examine the impact of these different geometric arrangements on the chemical partition of 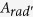. However, since the emission spectra, *A*_*rad*_, Ω_2_ and Ω_4_ have been measured for these complexes in the opposite TPPO configuration^6^,^40^, we will use these same values to arrive at the chemical partition for each of the two possible geometrical arrangements for enlightening purposes only. Tables S1 and S2 of the Supplementary Information contain information on the adjustments of the theoretical intensity parameters, unique for each of the geometrical arrangements, and the partition results, aggregated by ligand, for all three complexes considered.

**Figure 1:**
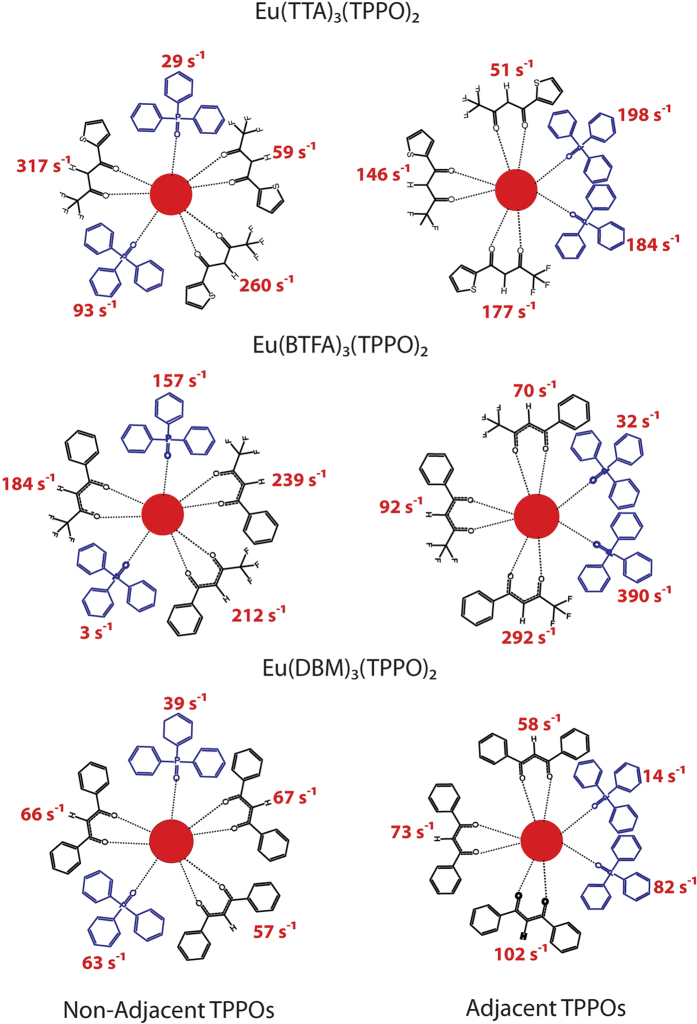
Chemical partition of A_rad′_ per ligand for two conformations of complexes of the type Eu(β-diketonate)_3_(TPPO)_2_: one with the two TPPOs non-adjacent, and the other with the two TPPOs adjacent to each other.

Figure 1 shows the chemical partition of the radiative decay rate 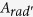 for each of the ligands coordinated to the metal ion for both cases of adjacent and opposite non-ionic ligands for all three TPPO complexes considered.

Figure 1 evidences the chemical nature of the partition because, now, 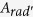 has been sliced into ligand terms that depend on the chemical nature of the ligands, as well as on their collective arrangements around the europium ion.

In an environment with three other identical ionic ligands, adjacent non-ionic ligands are less centrosymmetric than opposite ones. So, one would expect that adjacent same ligands should contribute more to the relaxation of the forbidding character of the ^5^D_0_ → ^7^F_J_ transitions (J = 2,4,6) than opposite ones. That is indeed the case for Eu(TTA)_3_(TPPO)_2,_ where the sum of terms of the adjacent TPPOs is 382 s^−1^, whereas for opposite TPPOs it is 122 s^−1^. Equivalent numbers for Eu(BTFA)_3_(TPPO)_2_ are 422 s^−1^ and 160 s^−1^, and for Eu(DBM)_3_(TPPO)_2_, they are 96 s^−1^ and 102 s^−1^, a more balanced situation which arises seemingly due to the more symmetric and bulky nature of DBM.

Conversely, the β-diketonate ligands are more centrosymmetric when the TPPOs are adjacent (two of them tend to occupy opposite axial-like positions) and therefore they should contribute less to the relaxation of the forbidding character of the ^5^D_0_ → ^7^F_J_ transitions (J = 2, 4, 6). On the other hand, the β-diketonate ligands are less centrosymmetric when the TPPOs are opposite, because in this case they tend to occupy planar trigonal-like positions, in which case they should contribute more to the relaxation of the forbidding character of the ^5^D_0_ → ^7^F_J_ transitions (J = 2, 4, 6). For Eu(TTA)_3_(TPPO)_2,_ the sum of the three β-diketonate terms for opposite TPPOs is 636 s^−1^, whereas for adjacent TPPOs it is 374 s^−1^. Equivalent numbers for Eu(BTFA)_3_(TPPO)_2_ are 635 s^−1^ and 454 s^−1^. For Eu(DBM)_3_(TPPO)_2_ the numbers are 190 s^−1^ and 233 s^−1^, which we again attribute to the bulky nature of DBM which is in itself the most symmetric of the β-diketonates used and makes the whole complex overall more centrosymmetric, severely reducing the value of A_rad_ to 335 s^−1^ when compared to the other two, which average 858 s^−1^.

### Applications of the chemical partition

Complexes Eu(TTA)_3_(TPPO)_2_ and Eu(BTFA)_3_(TPPO)_2_ had their geometries determined by crystallography and deposited in the Cambridge Structural Database, CSD^41^,^42^,^43^, with refcodes SABHIM and WIFWIR, respectively. In both cases, the non-ionic ligands appear opposite to each other. Recently, a theoretical determination of the thermodynamic properties of Eu(DBM)_3_(TPPO)_2_ also indicated that the opposite TPPO configuration should be the preferred one^40^. This study further extended the analysis for other non-ionic ligands and predicted that Eu(DBM)_3_(DBSO)_2_, and Eu(DBM)_3_(PTSO)_2_ should also display opposite non-ionic ligand configurations, where DBSO is dibenzyl sulfoxide and PTSO is p-tolyl sulfoxide. As a consequence, in the present article we assume that all complexes of the general formula Eu(β-diketonate)_3_(L)_2_ where L is a non-ionic ligand, will adopt opposite non-ionic ligand configurations. As before, all structural data have been computed by either RM1^22^, or, in a single case, by the Sparkle/PM3^18^ method - the choice of method followed the QDC acceptance criterion^27^.

Table 1 presents luminescence results for 9 different complexes of the general formula Eu(β-diketonate)_3_(L)_2_, radiative decay rates, both experimental (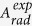) and calculated, (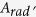), the latter one partitioned by ligands and summed up into ionic ligand (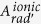) and non-ionic ligand (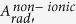) terms. Please remember that (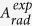) will always be larger than (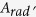) because 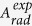 refers to all ^5^D_0_ → ^7^F_J_ transitions, while 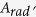 only adds up those with J = 2,4,6.

Examination of the average values in Table 1 reveals that the contribution of the non-ionic ligands to the triggering of luminescence decay by the excited europium trivalent ion is much smaller, in average 120 s^−1^, than the corresponding contribution of the ionic ligands, 544 s^−1^. That could lead to a misunderstanding that the non-ionic ligands, in these cases, are not too relevant to the luminescence phenomenon. However, their modest contribution to (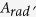) is seemingly due to that fact that they are opposite to each other, and, therefore, in a symmetric configuration with respect to the europium ion, which does not help to relax the Laporte’s rule.

Recently, our group introduced a simple strategy to boost three important luminescence properties of complexes of the general formula Eu(β-diketonate)_3_(L)_2_: the quantum yield, Φ, the emission efficiency η, and 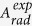, which was mathematically translated into the following conjecture^6^:


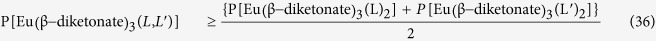


where P stands for either Φ, η, or 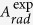 and L and L′ are different non-ionic ligands. This conjecture states that mixed non-ionic ligand complexes, Eu(β-diketonate)_3_(L,L′), should display larger luminescence properties when compared to the average of the same properties for repeating ligand complexes, Eu(β-diketonate)_3_(L)_2_ and Eu(β-diketonate)_3_(L′)_2_. This conjecture has already been proven experimentally for all combinations of the non-ionic ligands DBSO, TPPO, and PTSO, for all ternary europium complexes of TTA, BTFA, and DBM.

Table 2 displays 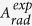, 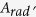, 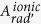 and 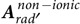 for the complexes with mixed non-ionic ligands. The role of the non-ionic ligands in the mixed-ligand complexes becomes clearer. Indeed, now the contribution of both different non-ionic ligands, to the triggering of luminescence decay, becomes accentuated to an average of 302 s^−1^, up from the average of 120 s^−1^ in the repeating non-ionic ligand complexes of Table 1. Once again, this behavior can be rationalized in terms of symmetry: the different non-ionic ligands are opposite to each other, rendering the situation considerably more asymmetric, thus much more capable of triggering the luminescent decay of the excited europium ion. On the other hand, the role of the ionic ligands remains, in average, unaffected. Indeed, the average of 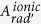 for the mixed non-ionic ligand complexes is 552 s^−1^, whereas in the repeating non-ionic ligand complexes it is 544 s^−1^, thus reinforcing the protagonist role of the non-ionic ligands in the luminescence boost.

So far, we have argued the usefulness of our chemical partition in terms of averages across complexes, which helped us understand some global aspects of the luminescence phenomenon.

We now turn to examine the usefulness of the chemical partition per complex. The conjecture, eq. (36), indicates that in order to obtain a more luminescent complex, of the type Eu(β-diketonate)_3_(L)_2_, one should synthesize complexes of the type Eu(β-diketonate)_3_(L,L′). However, the conjecture does not provide a hint of which pair combination of non-ionic ligands L,L′ one must choose to obtain the mixed non-ionic ligand complex of maximum 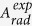. Actually, this is by no means trivial as exemplified by the case of Eu(DBM)_3_(L)_2_. Which pair of non-ionic ligands L,L′ will lead to the complex Eu(DBM)_3_(L,L′) with the largest 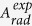
? If one naively decides to choose in terms of 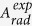 of Table 1, by picking the non-ionic ligands of the two complexes with the highest values of 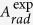: Eu(DBM)_3_(DBSO)_2_ and Eu(DBM)_3_(PTSO)_2_, one would synthesize complex Eu(DBM)_3_(DBSO,PTSO) and would be left with the mixed non-ionic DBM ternary complex with the smallest 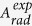. On the other hand, for the case of TTA ternary complexes, such a choice would lead to the correct complex, that is, to Eu(TTA)_3_(DBSO,TPPO). Clearly, choosing mixed non-ionic ligand combinations from 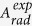 of repeating non-ionic ligand complexes is not correct, as extraneous factors seem to be playing a role in the different ligands synergy, factors that, as will be shown below, the chemical partition seems to unveil.

Since what one needs to choose is one pair of non-ionic ligands which will lead to the mixed ligand complex with the highest value of 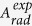, one must therefore simply look, instead, at the non-ionic ligand term 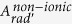 in the corresponding repeating ligand complexes in Table 1. Thus, for the before mentioned case of DBM ternary complexes, one would choose the two non-ionic ligands L, and L′, whose corresponding repeating non-ionic ligand complexes display the largest values of 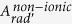: Eu(DBM)_3_(TPPO)_2_ and Eu(DBM)_3_(DBSO)_2_, respectively, 102 s^−1^, and 59 s^−1^, leading to complex Eu(DBM)_3_(DBSO,TPPO). Indeed, this complex has the largest 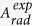 of 652 s^−1^. Furthermore, one can use the same reasoning to arrive at the DBM ternary complex with the next larger 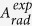, where two possibilities exist: Eu(DBM)_3_(PTSO,TPPO) and Eu(DBM)_3_(DBSO,PTSO). The corresponding pair of largest 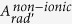 that are left are 102 s^−1^ for Eu(DBM)_3_(TPPO)_2_ and 29 s^−1^ for Eu(DBM)_3_(PTSO)_2_, leading to complex Eu(DBM)_3_(PTSO,TPPO), which, indeed, is the next best complex, with an 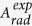 of 572 s^−1^. Finally, the last one left is complex Eu(DBM)_3_(DBSO,PTSO), with an 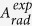 of 540 s^−1^.

Undoubtedly, for the case of ternary complexes of DBM, usage of the chemical partition allowed us to perfectly order, in an *a priori* way, the pair combinations of non-ionic ligands in terms of their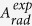, a single outcome out of six possibilities.

**Figure 2:**
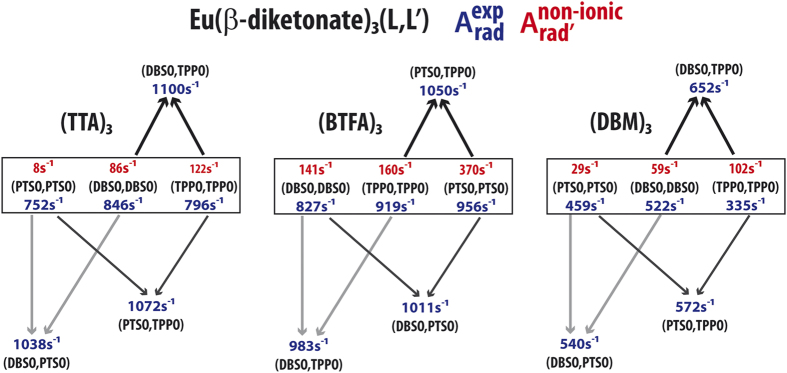
Choice (represented by the arrows) of pairs of non-ionic ligands (in parenthesis) from the values (in red) of 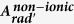 of the repeating non-ionic ligand complexes (inside the rectangles), perfectly orders all mixed non-ionic ligand complexes in terms of their 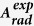 values (in blue).

Let us now apply the same strategy to the other ternary complexes of TTA and BTFA. The overall situation is present in pictorial format in Fig. 2, which shows that choosing non-ionic ligands by their 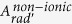 terms in non-ionic repeating ligand complexes also perfectly orders all mixed non-ionic ligand complexes in terms of their 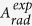 values for the remaining cases of TTA and BTFA ternary complexes.

Overall, the chemical partition predicted, in an *a priori* manner, a single joint outcome out of 216 possibilities, which, if it were by chance, would have a probability of only 0.46% of occurrence.

## Conclusions

For the first time, a molecular global property related to luminescence, the radiative decay rate of europium complexes, is partitioned into ligand contributions. Such a novel approach gives rise to possible chemical interpretations of the effect of each ligand and their interactions on the luminescence phenomenon, allowing for the design of cutting edge compounds with enhanced brightness upon ultra-violet illumination.

As a demonstration of an application made only possible with the chemical partition, we address the case of repeating non-ionic ligand ternary complexes of europium(III) with DBM, TTA, and BTFA. We show that the chemical partition allows us to perfectly order, in an *a priori* way, the non-obvious pair combinations of non-ionic ligands that led to mixed-ligand compounds with larger values of A_rad_.

Our new chemical partition has been implemented in the LUMPAC software, which is freely available^12^.

## Additional Information

**How to cite this article**: Lima, N. B. D. *et al.* Chemical Partition of the Radiative Decay Rate of Luminescence of Europium Complexes. *Sci. Rep.* **6**, 21204; doi: 10.1038/srep21204 (2016).

## Supplementary Material

Supplementary Information

## Figures and Tables

**Table 1:** Radiative decay rates 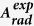 and 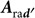 as well as the ionic and non-ionic partitions of 
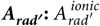
 and 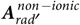.

Complex	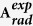 (s^−1^)	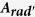 (s^−1^)	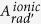 (s^−1^)	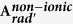 (s^−1^)
Eu(TTA)_3_(DBSO)_2_	846	806	721	86
Eu(TTA)_3_(TPPO)_2_	796	757	635	122
Eu(TTA)_3_(PTSO)_2_	752	718	709	8
Eu(BTFA)_3_(DBSO)_2_	827	792	651	141
Eu(BTFA)_3_(TPPO)_2_	919	795	635	160
Eu(BTFA)_3_(PTSO)_2_	956	919	550	370
Eu(DBM)_3_(DBSO)_2_	522	477	418	59
Eu(DBM)_3_(TPPO)_2_^a^	335	292	190	102
Eu(DBM)_3_(PTSO)_2_	459	413	383	29
**Averages**	**712**	**663**	**544**	**120**

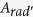
 corresponds to the transitions from ^5^D_0_ to ^7^F_2_, ^7^F_4_, and ^7^F_6_, and is therefore always smaller than 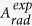 which, in addition, also includes the transitions to ^7^F_0_, ^7^F_1_, ^7^F_3_, and ^7^F_5_. The 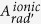
partition comprises the terms for each of the three identical β-diketonates The 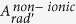 partition comprises the terms for each of the two identical non-ionic ligands. Geometries were optimized and the chemical partitions were calculated with the RM1 model (except where otherwise indicated).

^a^Geometry was optimized and the chemical partition was calculated with Sparkle/PM3.

**Table 2:** Radiative decay rates 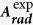 and 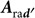 as well as the ionic and non-ionic partitions of 
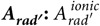
 and 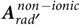.

Complex	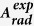 (s^−1^)	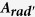 (s^−1^)	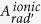 (s^−1^)	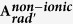 (s^−1^)
Eu(TTA)_3_(DBSO,TPPO)	1100	1061	926	135
Eu(TTA)_3_(PTSO,TPPO)	1072	1023	459	564
Eu(TTA)_3_(DBSO,PTSO)	1038	1004	626	379
Eu(BTFA)_3_(DBSO,TPPO)^a^	983	949	440	509
Eu(BTFA)_3_(PTSO,TPPO)	1050	1036	927	108
Eu(BTFA)_3_(DBSO,PTSO)	1011	980	219	761
Eu(DBM)_3_(DBSO,TPPO)	652	609	566	43
Eu(DBM)_3_(PTSO,TPPO)	572	528	410	117
Eu(DBM)_3_(DBSO,PTSO)	540	496	391	105
**Averages**	**891**	**854**	**552**	**302**

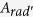
 corresponds to the transitions from ^5^D_0_ to ^7^F_2_, ^7^F_4_, and ^7^F_6_, and is therefore always smaller than 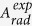 which, in addition, also includes the transitions to ^7^F_0_, ^7^F_1_, ^7^F_3_, and ^7^F_5_. The 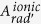 partition comprises the terms for each of the three identical β-diketonates The 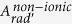 partition comprises the terms for each of the two non-ionic ligands. Geometries were optimized and the chemical partitions were calculated with the RM1 model (except where otherwise indicated).

^a^Geometry was optimized and the chemical partition was calculated with Sparkle/RM1.

## References

[b1] Horrocks W. D. & Sudnick D. R. Lanthanide ion luminescence probes of the structure of biological macromolecules. Acc. Chem. Res. 14, 384–392 (1981).

[b2] Sabbatini N., Guardigli M. & Lehn J. M. Luminescent Lanthanide Complexes as Photochemical Supramolecular Devices. Coord. Chem. Rev. 123, 201–228 (1993).

[b3] Thejokalyani N. & Dhoble S. J. Novel approaches for energy efficient solid state lighting by RGB organic light emitting diodes–A review. Renew. Sust. Energ. Rev. 32, 448–467 (2014).

[b4] Kalyani N. T. & Dhoble S. J. Organic light emitting diodes: Energy saving lighting technology-A review. Renew. Sust. Energ. Rev. 16, 2696–2723 (2012).

[b5] Lehn J. M. Perspectives in Supramolecular Chemistry - from Molecular Recognition Towards Molecular Information-Processing and Self-Organization. Angew. Chem.-Int. Edit. Engl. 29, 1304–1319 (1990).

[b6] Lima N. B. D., Gonçalves S. M. C., Júnior S. A. & Simas A. M. A Comprehensive Strategy to Boost the Quantum Yield of Luminescence of Europium Complexes. Sci. Rep. 3, 2395 (2013). 23928866 10.1038/srep02395 PMC3738935

[b7] Diniz J. R. *et al.* Water-Soluble Tb^3+^ and Eu^3+^ Complexes with Ionophilic (Ionically Tagged) Ligands as Fluorescence Imaging Probes. Inorg. Chem. 52, 10199–10205 (2013). 23944354 10.1021/ic4017678

[b8] Hagan A. K. & Zuchner T. Lanthanide-based time-resolved luminescence immunoassays. Anal. Bioanal. Chem. 400, 2847–2864 (2011). 21556751 10.1007/s00216-011-5047-7 PMC3102841

[b9] Kimura H. *et al.* Quantitative evaluation of time-resolved fluorescence microscopy using a new europium label: Application to immunofluorescence imaging of nitrotyrosine in kidneys. Anal. Biochem. 372, 119–121 (2008). 17964525 10.1016/j.ab.2007.09.016

[b10] Bunzli J. C. G. & Piguet C. Taking advantage of luminescent lanthanide ions. Chem. Soc. Rev. 34, 1048–1077 (2005). 16284671 10.1039/b406082m

[b11] Armelao L. *et al.* Design of luminescent lanthanide complexes: From molecules to highly efficient photo-emitting materials. Coord. Chem. Rev. 254, 487–505 (2010).

[b12] Dutra J. D. L., Bispo T. D. & Freire R. O. LUMPAC lanthanide luminescence software: Efficient and user friendly. J. Comput. Chem. 35, 772–775 (2014). 24532191 10.1002/jcc.23542

[b13] Albuquerque R. Q., Da Costa N. B. & Freire R. O. Design of new highly luminescent Tb^3+^ complexes using theoretical combinatorial chemistry. J. Lumin. 131, 2487–2491 (2011).

[b14] Dutra J. D. L., Gimenez I. F., da Costa N. B. & Freire R. O. Theoretical design of highly luminescent europium (III) complexes: A factorial study. J. Photoch. Photobio. A 217, 389–394 (2011).

[b15] Freire R. O., Silva F. R. G. E., Rodrigues M. O., de Mesquita M. E. & Junior N. B. D. Design of europium(III) complexes with high quantum yield. J. Mol. Model. 12, 16–23 (2005). 16044288 10.1007/s00894-005-0280-7

[b16] Freire R. O., Albuquerque R. Q., Junior S. A., Rocha G. B. & de Mesquita M. E. On the use of combinatory chemistry to the design of new luminescent Eu^3+^ complexes. Chem. Phys. Lett. 405, 123–126 (2005).

[b17] Freire R. O., Rocha G. B. & Simas A. M. Sparkle model for the calculation of lanthanide complexes: AM1 parameters for Eu(III), Gd(III), and Tb(III). Inorg. Chem. 44, 3299–3310 (2005). 15847440 10.1021/ic048530+

[b18] Freire R. O., Rocha G. B. & Simas A. M. Sparkle/PM3 for the Modeling of Europium(III), Gadolinium(III), and Terbium(III) Complexes. J. Brazil Chem. Soc. 20, 1638–1645 (2009).

[b19] Freire R. O. & Simas A. M. Sparkle/PM6 Parameters for all Lanthanide Trications from La(III) to Lu(III). J. Chem. Theory Comput. 6, 2019–2023 (2010). 26615930 10.1021/ct100192c

[b20] Dutra J. D. L. *et al.* Sparkle/PM7 Lanthanide Parameters for the Modeling of Complexes and Materials. J. Chem. Theory Comput. 9, 3333–3341 (2013). 24163641 10.1021/ct301012h PMC3806451

[b21] Filho M. A. M., Dutra J. D. L., Rocha G. B., Freire R. O. & Simas A. M. Sparkle/RM1 parameters for the semiempirical quantum chemical calculation of lanthanide complexes. RSC Adv. 3, 16747–16755 (2013).

[b22] Filho M. A. *et al.* RM1 Model for the Prediction of Geometries of Complexes of the Trications of Eu, Gd, and Tb. J. Chem. Theory Comput. 10, 3031–3037 (2014). 26588274 10.1021/ct400909w

[b23] Filho M. A. M., Dutra J. D. L., Rocha G. B., Simas A. M. & Freire R. O. Semiempirical Quantum Chemistry Model for the Lanthanides: RM1 (Recife Model 1) Parameters for Dysprosium, Holmium and Erbium. Plos One 9 **(1)**, e86376 (2014). 24497945 10.1371/journal.pone.0086376 PMC3908927

[b24] Filho M. A. M., Dutra J. D. L., Rocha G. B., Simas A. M. & Freire R. O. RM1 modeling of neodymium, promethium, and samarium coordination compounds. RSC Adv. 5, 12403–12408 (2015).

[b25] Dutra J. D. L., Filho M. A. M., Rocha G. B., Simas A. M. & Freire R. O. RM1 Semiempirical Quantum Chemistry: Parameters for Trivalent Lanthanum, Cerium and Praseodymium. PLoS One 10, e0124372 (2015). 26132289 10.1371/journal.pone.0124372 PMC4489505

[b26] MOPAC2012, Stewart, J.J.P., Stewart Computational Chemistry, Colorado Springs, CO, USA, 2012 (http://OpenMOPAC.net).

[b27] Dutra J. D. L., Lima N. B. D., Freire R. O. & Simas A. M. Europium Luminescence: Electronic Densities and Superdelocalizabilities for a Unique Adjustment of Theoretical Intensity Parameters. Sci. Rep. 5, 13695 (2015). 26329420 10.1038/srep13695 PMC4557129

[b28] de Sa G. F. *et al.* Spectroscopic properties and design of highly luminescent lanthanide coordination complexes. Coord. Chem. Rev. 196, 165–195 (2000).

[b29] Carnall W. T., Crosswhite H. & Crosswhite H. M. Energy level structure and transition probabilities of the trivalent lanthanides in LaF_3_, in Argonne National Laboratory Report (1978). Available at: http://www.osti.gov/scitech/biblio/6417825. (Accessed: 29^th^ October 2015).

[b30] Binnemans K., De Leebeeck H., Görller-Walrand C. & Adam J. L. Visualisation of the reliability of Judd–Ofelt intensity parameters by graphical simulation of the absorption spectrum. Chem. Phys. Lett. 303, 76–80 (1999).

[b31] Weber M. J., Varitimo T. E. & Matsinge B. H. Optical Intensities of Rare-Earth Ions in Yttrium Orthoaluminate. Phys. Rev. B 8, 47–53 (1973).

[b32] Judd B. R. Optical Absorption Intensities of Rare-Earth Ions. Phys. Rev. 127, 750–761 (1962).

[b33] Ofelt G. S. Intensities of Crystal Spectra of Rare-Earth Ions. J. Chem. Phys. 37, 511–520 (1962).

[b34] Freeman A. J. & Desclaux J. P. Dirac-Fock Studies of Some Electronic Properties of Rare-Earth Ions. J. Magn. Magn. Mater. 12, 11–21 (1979).

[b35] Malta O. L., Ribeiro S. J. L., Faucher M. & Porcher P. Theoretical Intensities of 4f-4f Transitions between Stark Levels of the Eu^3+^ Ion in Crystals. J. Phys. Chem. Solids 52, 587–593 (1991).

[b36] Malta O. L. & Silva F. R. G. E. A theoretical approach to intramolecular energy transfer and emission quantum yields in coordination compounds of rare earth ions. Spectrochim Acta A 54, 1593–1599 (1998).

[b37] Malta O. L. A Simple Overlap Model in Lanthanide Crystal-Field Theory. Chem. Phys. Lett. 87, 27–29 (1982).

[b38] Malta O. L. Theoretical Crystal-Field Parameters for the YOCl:Eu^3+^ System. A Simple Overlap Model. Chem. Phys. Lett. 88, 353–356 (1982).

[b39] Bethe H. Termaufspaltung in Kristallen. Ann. Physik 395, 133–208 (1929).

[b40] Lima N. B. D., Silva A. I. S., Gonçalves S. M. C. & Simas A. M. Synthesis of mixed ligand europium complexes: Verification of predicted luminescence intensification. J. Lumin. in press (2015).

[b41] Allen F. H. The Cambridge Structural Database: a quarter of a million crystal structures and rising. Acta Crystallogr. B 58, 380–388 (2002). 12037359 10.1107/s0108768102003890

[b42] Allen F. H. & Motherwell W. D. S. Applications of the Cambridge Structural Database in organic chemistry and crystal chemistry. Acta Crystallogr. B 58, 407–422 (2002). 12037362 10.1107/s0108768102004895

[b43] Bruno I. J. *et al.* New software for searching the Cambridge Structural Database and visualizing crystal structures. Acta Crystallogr. B 58, 389–397 (2002). 12037360 10.1107/s0108768102003324

