# Supplementary material for: Chemical Partition of the Radiative Decay Rate of Luminescence of Europium Complexes
Source: Sci Rep. 2016 Feb 19;6:21204. doi: 10.1038/srep21204 (PMC4759827; doi:10.1038/srep21204)
Supplement: Supplementary Information [file srep21204-s1.pdf]

Supplementary Information

Chemical Partition of the Radiative Decay Rate of Luminescence of Europium Complexes

*Nathalia B. D. Lima[a], José Diogo L. Dutra[a,b], Simone M. C. Gonçalves[a], Ricardo O. Freire[b], and Alfredo M. Simas[a]**

[a]Departamento de Química Fundamental, CCEN, UFPE, 50590-470 - Recife, PE, Brazil.

[b]Pople Computational Chemistry Laboratory, Departamento de Química, CCET, UFS, 49100-000 - São Cristóvão, SE, Brazil.

* Corresponding author: E-mail: simas@ufpe.br
Tel. +55 81 2126-8434

List of Tables

Table S1. Fitted Q, D, and C values for all complexes studied, with electronic densities and electrophilic superdelocalizabilities[1], together with calculated and experimental Ω_λ values for the complexes of the general formula Eu(β-diketonate)$_3$(TPPO)$_2$ with the RM1 model (except where otherwise indicated), where β-diketonate stands for the ionic ligands TTA, BTFA, and DBM.† 4

Table S2. Fitted Q, D, and C values for all complexes studied, with electronic densities and electrophilic superdelocalizabilities[1], together with calculated and experimental Ω_λ values for the complexes of the general formula Eu(β-diketonate)$_3$(L)$_2$ with the RM1 model (except where otherwise indicated), where β-diketonate stands for the ionic ligands TTA, BTFA, and DBM; and L stands for the non-ionic ligands TPPO, DBSO, and PTSO. † 5

Table S3. Fitted Q, D, and C values for all complexes studied, with electronic densities and electrophilic superdelocalizabilities[1], together with calculated and experimental Ω_λ values for the complexes of the general formula Eu(β-diketonate)$_3$(L,L') with the RM1 model (except where otherwise indicated), where β-diketonate stands for the ionic ligands TTA, BTFA, and DBM; and L and L' stand for the non-ionic ligands TPPO, DBSO, and PTSO. † 6

Table S4. Radiative decay rates A_{rad}^{exp} and $A_{rad'}$, as well as the ionic and non-ionic partitions of $A_{rad'}$: $A_{rad'}^{ionic}$ and $A_{\mathrm{rad}'}^{\mathrm{non-ionic}}$. $A_{rad'}$ corresponds to the transitions from $^5D_0$ to $^7F_2$, $^7F_4$, and $^7F_6$, and is therefore always smaller than A_{rad}^{exp} which, in addition, also includes the transitions to $^7F_0$, $^7F_1$, $^7F_3$, and $^7F_5$. The $A_{rad'}^{ionic}$ partition comprises the terms for each of the three identical β-diketonates, ordered into maximum (Max), medium (Med) and minimum (Min) values. The $A_{rad'}^{non-ionic}$ partition comprises the terms for each of the two identical non-ionic ligands, ordered into maximum (Max), and minimum (Min) values. Geometries were optimized and the chemical partitions were calculated with the RM1 model (except where otherwise indicated). 7

Table S5. Radiative decay rates A_{rad}^{exp} and $A_{rad'}$, as well as the ionic and non-ionic partitions of $A_{rad'}$: $A_{rad'}^{ionic}$ and $A_{rad'}^{non-ionic}$. $A_{rad'}$ corresponds to the transitions from $^5D_0$ to $^7F_2$, $^7F_4$, and $^7F_6$, and is therefore always smaller than A_{rad}^{exp} which, in addition, also includes the transitions to $^7F_0$, $^7F_1$, $^7F_3$, and $^7F_5$. The $A_{rad'}^{ionic}$ partition comprises the terms for each of the three identical β-diketonates, ordered into maximum (Max), medium (Med) and minimum (Min) values. The $A_{rad'}^{non-ionic}$ partition comprises the terms for each of the two identical non-ionic ligands, ordered into maximum (Max), and minimum (Min) values. Geometries were optimized and the chemical partitions were calculated with the RM1 model (except where otherwise indicated). 8

Table S6. Radiative decay rates A_{rad}^{exp} and $A_{rad'}$, as well as the ionic and non-ionic partitions of $A_{rad'}$: $A_{rad'}^{ionic}$ and $A_{rad'}^{non-ionic}$. $A_{rad'}$ corresponds to the transitions from $^5D_0$ to $^7F_2$, $^7F_4$, and $^7F_6$, and is therefore always smaller than A_{rad}^{exp} which, in addition, also includes the transitions to $^7F_0$, $^7F_1$, $^7F_3$, and $^7F_5$. The $A_{rad'}^{ionic}$ partition comprises the terms for each of the three identical β-diketonates, ordered into maximum (Max), medium (Med) and minimum (Min) values. The $A_{rad'}^{non-ionic}$ partition is comprised of terms corresponding to each of the non-ionic ligands, as

indicated. Geometries were optimized and the chemical partitions were calculated with the RM1 model (except where otherwise indicated). 9

Table S7. Spherical coordinates of all atoms belonging to the coordination polyhedron of the complexes of general formula $Eu(BTFA)_3(L)_2$ and $Eu(BTFA)_3(L,L')$, optimized with the RM1 model (except where otherwise indicated), where L and L' stand for the non-ionic ligands TPPO, DBSO, and PTSO 10

Table S8. Spherical coordinates of all atoms belonging to the coordination polyhedron of the complexes of general formula $Eu(TTA)_3(L)_2$ and $Eu(TTA)_3(L,L')$ optimized with the RM1 model, where L and L' stand for the non-ionic ligands TPPO, DBSO, and PTSO..................... 13

Table S9. Spherical coordinates of all atoms belonging to the coordination polyhedron of the complexes of general formula $Eu(DBM)_3(L)_2$ and $Eu(DBM)_3(L,L')$ optimized with the RM1 model (except where otherwise indicated), where β-diketonate stands for the ionic ligands DBM; and L and L' stand for the non-ionic ligands TPPO, DBSO, and PTSO. 16

Table S1. Fitted Q, D, and C values for all complexes studied, with electronic densities and electrophilic superdelocalizabilities[1], together with calculated and experimental Ω_λ values for the complexes of the general formula Eu(β-diketonate)$_3$(TPPO)$_2$ with the RM1 model (except where otherwise indicated), where β-diketonate stands for the ionic ligands TTA, BTFA, and DBM.†

Complex	Q	D	C	D/C	Ω_2^{calc}	Ω_2^{exp}	Ω_4^{calc}	Ω_4^{exp}	Ω_6^{calc}
Eu(TTA)$_3$(TPPO)$_{2,\text{Non-Adj}}$	0.145	42.9	20.8	2.06	24.7	24.8	6.53	6.47	0.323
Eu(TTA)$_3$(TPPO)$_{2,\text{Adj}}$	0.0871	32.9	17.7	1.86	24.8	24.8	6.46	6.47	0.177
Eu(BTFA)$_3$(TPPO)$_{2,\text{Non-Adj}}$	0.0045	81.7	43.3	1.89	28.7	28.6	1.41	7.73	0.179
Eu(BTFA)$_3$(TPPO)$_{2,\text{Adj}}$	0.0759	44.8	22.0	2.04	28.6	28.6	7.74	7.73	0.212
Eu(DBM)$_3$(TPPO)$_{2,\text{Non-Adj}}$ (a)	0.0673	17.4	11.9	1.46	6.92	6.92	7.69	7.69	0.137
Eu(DBM)$_3$(TPPO)$_{2,\text{Adj}}$	0.0835	19.9	11.6	1.71	6.91	6.92	7.70	7.69	0.174

†Units are: *Q* (au^{-1}); *D* ($au^{-1}\cdot Å^3$); *C* ($Å^3$); *D/C* (au^{-1}); Ω_λ ($10^{-20} cm^2$).

(a) Geometry was optimized and the chemical partition was calculated with Sparkle/PM3.

Table S2. Fitted Q, D, and C values for all complexes studied, with electronic densities and electrophilic superdelocalizabilities[1], together with calculated and experimental Ω_λ values for the complexes of the general formula Eu(β-diketonate)$_3$(L)$_2$ with the RM1 model (except where otherwise indicated), where β-diketonate stands for the ionic ligands TTA, BTFA, and DBM; and L stands for the non-ionic ligands TPPO, DBSO, and PTSO.[†]

Complex	Q	D	C	D/C	Ω_2^{calc}	Ω_2^{exp}	Ω_4^{calc}	Ω_4^{exp}	Ω_6^{calc}
Eu(TTA)$_3$(DBSO)$_{2,\text{Non-Adj}}$	0.0972	29.9	14.6	2.04	26.3	26.3	7.16	7.07	0.290
Eu(TTA)$_3$(PTSO)$_{2,\text{Non-Adj}}$	0.0416	28.2	13.5	2.09	23.5	23.5	6.11	6.11	0.259
Eu(TTA)$_3$(TPPO)$_{2,\text{Non-Adj}}$	0.145	42.9	20.8	2.06	24.7	24.8	6.53	6.47	0.323
Eu(BTFA)$_3$(DBSO)$_{2,\text{Non-Adj}}$	0.242	43.1	19.9	2.16	27.3	27.5	3.95	3.02	0.981
Eu(BTFA)$_3$(PTSO)$_{2,\text{Non-Adj}}$	0.254	44.0	21.0	2.10	30.4	30.6	7.18	6.62	1.01
Eu(BTFA)$_3$(TPPO)$_{2,\text{Non-Adj}}$	0.0045	81.7	43.3	1.89	28.7	28.6	1.41	7.73	0.179
Eu(DBM)$_3$(DBSO)$_{2,\text{Non-Adj}}$	0.0760	19.3	11.7	1.65	14.5	14.5	6.32	6.33	0.158
Eu(DBM)$_3$(PTSO)$_{2,\text{Non-Adj}}$	0.0593	20.4	11.4	1.79	12.5	12.5	5.56	5.57	0.163
Eu(DBM)$_3$(TPPO)$_{2,\text{Non-Adj}}$[(a)]	0.0673	17.4	11.9	1.46	6.92	6.92	7.69	7.69	0.137

[†]Units are: Q (au^{-1}); D (au^{-1}·Å^3); C (Å^3); D/C (au^{-1}); Ω_λ (10^{-20}cm^2).

[(a)] Geometry was optimized and the chemical partition was calculated with Sparkle/PM3.

Table S3. Fitted Q, D, and C values for all complexes studied, with electronic densities and electrophilic superdelocalizabilities[1], together with calculated and experimental Ω_λ values for the complexes of the general formula Eu(β-diketonate)$_3$(L,L’) with the RM1 model (except where otherwise indicated), where β-diketonate stands for the ionic ligands TTA, BTFA, and DBM; and L and L’ stand for the non-ionic ligands TPPO, DBSO, and PTSO. †

Complex	Q	D	C	D/C	Ω_2^{calc}	Ω_2^{exp}	Ω_4^{calc}	Ω_4^{exp}	Ω_6^{calc}
Eu(TTA)$_3$(DBSO,TPPO)$_{\text{Non-Adj}}$	0.158	47.6	23.0	2.07	36.3	36.3	5.97	5.92	0.383
Eu(TTA)$_3$(PTSO,TPPO)$_{\text{Non-Adj}}$	0.0025	67.1	33.3	2.02	35.0	35.1	5.65	6.19	0.402
Eu(TTA)$_3$(DBSO,PTSO)$_{\text{Non-Adj}}$	0.277	35.4	16.3	2.18	33.2	33.6	7.86	6.43	1.64
Eu(BTFA)$_3$(DBSO,TPPO)$_{\text{Non-Adj}}$[(a)]	0.280	45.8	24.1	1.90	31.8	32.5	6.43	4.10	0.940
Eu(BTFA)$_3$(PTSO,TPPO)$_{\text{Non-Adj}}$	0.290	38.8	18.2	2.13	34.0	34.6	8.63	6.23	1.82
Eu(BTFA)$_3$(DBSO,PTSO)$_{\text{Non-Adj}}$	0.297	79.4	34.5	2.30	32.8	33.2	6.83	5.40	1.34
Eu(DBM)$_3$(DBSO,TPPO)$_{\text{Non-Adj}}$	0.248	33.8	18.4	1.84	18.7	19.0	7.54	7.11	0.850
Eu(DBM)$_3$(PTSO,TPPO)$_{\text{Non-Adj}}$	0.107	38.2	18.8	2.03	15.0	15.0	9.03	9.01	0.350
Eu(DBM)$_3$(DBSO,PTSO)$_{\text{Non-Adj}}$	0.0647	28.7	15.4	1.87	14.3	14.3	8.08	8.08	0.224

†Units are: Q (au^{-1}); D (au^{-1}·Å^3); C (Å^3); D/C (au^{-1}); Ω_λ (10^{-20}cm^2).

[(a)] Geometry was optimized and the chemical partition was calculated with Sparkle/RM1.

Table S4. Radiative decay rates A_{rad}^{exp} and $A_{rad'}$, as well as the ionic and non-ionic partitions of $A_{rad'}$: $A_{rad'}^{ionic}$ and $A_{rad'}^{non-ionic}$. $A_{rad'}$ corresponds to the transitions from $^5D_0$ to $^7F_2$, $^7F_4$, and $^7F_6$, and is therefore always smaller than A_{rad}^{exp} which, in addition, also includes the transitions to $^7F_0$, $^7F_1$, $^7F_3$, and $^7F_5$. The $A_{rad'}^{ionic}$ partition comprises the terms for each of the three identical β-diketonates, ordered into maximum (Max), medium (Med) and minimum (Min) values. The $A_{rad'}^{non-ionic}$ partition comprises the terms for each of the two identical non-ionic ligands, ordered into maximum (Max), and minimum (Min) values. Geometries were optimized and the chemical partitions were calculated with the RM1 model (except where otherwise indicated).

Complex	A_{rad}^{exp}(s^{-1})	$A_{rad'}$(s^{-1})	$A_{rad'}^{ionic}$ (s^{-1})			$A_{rad'}^{non-ionic}$(s^{-1})	
			Max	Med	Min	Max	Min
$Eu(TTA)_3(TPPO)_{2,Non\text{-}Adj}$	796	757	317 (42%)	260 (34%)	59 (8%)	93 (12%)	29 (4%)
$Eu(TTA)_3(TPPO)_{2,Adj}$	796	757	177 (23%)	146 (19%)	51 (7%)	198 (26%)	184 (24%)
$Eu(BTFA)_3(TPPO)_{2,Non\text{-}Adj}$	919	795	239 (30%)	212 (27%)	184 (23%)	157 (20%)	3 (0%)
$Eu(BTFA)_3(TPPO)_{2,Adj}$	919	877	292 (33%)	92 (11%)	70 (8%)	390 (45%)	72 (4%)
$Eu(DBM)_3(TPPO)_{2,Non\text{-}Adj}$[(a)]	335	292	67 (23%)	66 (23%)	57 (20%)	63 (22%)	39 (13%)
$Eu(DBM)_3(TPPO)_{2,Adj}$	335	328	102 (31%)	73 (22%)	58 (18%)	82 (25%)	14 (4%)

[(a)] Geometry was optimized and the chemical partition was calculated with Sparkle/PM3.

Table S5. Radiative decay rates A_{rad}^{exp} and $A_{rad'}$, as well as the ionic and non-ionic partitions of $A_{rad'}$: $A_{rad'}^{ionic}$ and $A_{rad'}^{non-ionic}$. $A_{rad'}$ corresponds to the transitions from $^5D_0$ to $^7F_2$, $^7F_4$, and $^7F_6$, and is therefore always smaller than A_{rad}^{exp} which, in addition, also includes the transitions to $^7F_0$, $^7F_1$, $^7F_3$, and $^7F_5$. The $A_{rad'}^{ionic}$ partition comprises the terms for each of the three identical β-diketonates, ordered into maximum (Max), medium (Med) and minimum (Min) values. The $A_{rad'}^{non-ionic}$ partition comprises the terms for each of the two identical non-ionic ligands, ordered into maximum (Max), and minimum (Min) values. Geometries were optimized and the chemical partitions were calculated with the RM1 model (except where otherwise indicated).

Complex	A_{rad}^{exp}(s^{-1})	$A_{rad'}$(s^{-1})	$A_{rad'}^{ionic}$ (s^{-1})			$A_{rad'}^{non-ionic}$(s^{-1})	
			Max	Med	Min	Max	Min
$Eu(TTA)_3(DBSO)_{2,Non-Adj}$	846	806	525 (65%)	133 (17%)	62 (8%)	73 (9%)	12 (2%)
$Eu(TTA)_3(PTSO)_{2,Non-Adj}$	846	806	511 (71%)	111 (15%)	88 (12%)	6 (1%)	3 (0%)
$Eu(TTA)_3(TPPO)_{2,Non-Adj}$	796	757	317 (42%)	260 (34%)	59 (8%)	93 (12%)	29 (4%)
$Eu(BTFA)_3(DBSO)_{2,Non-Adj}$	827	792	413 (52%)	166 (21%)	72 (9%)	113 (14%)	27 (3%)
$Eu(BTFA)_3(PTSO)_{2,Non-Adj}$	956	919	330 (36%)	181 (20%)	39 (4%)	348 (38%)	22 (2%)
$Eu(BTFA)_3(TPPO)_{2,Non-Adj}$	919	795	239 (30%)	212 (27%)	184 (23%)	157 (20%)	3 (0%)
$Eu(DBM)_3(DBSO)_{2,Non-Adj}$	522	477	247 (52%)	91 (19%)	80 (17%)	35 (7%)	24 (5%)
$Eu(DBM)_3(PTSO)_{2,Non-Adj}$	459	413	156 (38%)	134 (32%)	93 (23%)	18 (4%)	11 (3%)
$Eu(DBM)_3(TPPO)_{2,Non-Adj}$[(a)]	335	292	67 (23%)	66 (23%)	57 (20%)	63 (22%)	39 (13%)

[(a)] Geometry was optimized and the chemical partition was calculated with Sparkle/PM3.

Table S6. Radiative decay rates A_{rad}^{exp} and $A_{rad'}$, as well as the ionic and non-ionic partitions of $A_{rad'}$: $A_{rad'}^{ionic}$ and $A_{rad'}^{non-ionic}$. $A_{rad'}$ corresponds to the transitions from $^5D_0$ to $^7F_2$, $^7F_4$, and $^7F_6$, and is therefore always smaller than A_{rad}^{exp} which, in addition, also includes the transitions to $^7F_0$, $^7F_1$, $^7F_3$, and $^7F_5$. The $A_{rad'}^{ionic}$ partition comprises the terms for each of the three identical β-diketonates, ordered into maximum (Max), medium (Med) and minimum (Min) values. The $A_{rad'}^{non-ionic}$ partition is comprised of terms corresponding to each of the non-ionic ligands, as indicated. Geometries were optimized and the chemical partitions were calculated with the RM1 model (except where otherwise indicated).

Complex	A_{rad}^{exp} (s^{-1})	$A_{rad'}$ (s^{-1})	$A_{rad'}^{ionic}$ (s^{-1})			$A_{rad'}^{non-ionic}$ (s^{-1})		
			Max	Min	Med	DBSO	PTSO	TPPO
$Eu(TTA)_3$(DBSO,TPPO) Non-Adj	1100	1061	470 (44%)	322 (30%)	134 (10%)	30 (3%)	------	105 (10%)
$Eu(TTA)_3$(PTSO,TPPO) Non-Adj	1072	1023	216 (21%)	129 (13%)	115 (11%)	------	536 (52%)	28 (3%)
$Eu(TTA)_3$(DBSO,PTSO) Non-Adj	1038	1004	392 (39%)	179 (18%)	55 (6%)	364 (36%)	14 (1%)	------
$Eu(BTFA)_3$(DBSO,TPPO) Non-Adj[(a)]	983	949	216 (23%)	113 (12%)	110 (12%)	416 (44%)	------	94 (10%)
$Eu(BTFA)_3$(PTSO,TPPO) Non-Adj	1050	1036	456 (44%)	409 (39%)	62 (6%)	------	54 (5%)	54 (5%)
$Eu(BTFA)_3$(DBSO,PTSO) Non-Adj	1011	980	106 (11%)	90 (9%)	22 (2%)	261 (27%)	501 (51%)	------
$Eu(DBM)_3$(DBSO,PTSO) Non-Adj	652	609	335 (55%)	134 (22%)	97 (16%)	36 (6%)	------	7 (1%)
$Eu(DBM)_3$(PTSO,TPPO) Non-Adj	572	528	275 (52%)	103 (20%)	32 (6%)	------	79 (7%)	79 (15%)
$Eu(DBM)_3$(DBSO,PTSO) Non-Adj	540	496	245 (49%)	78 (16%)	68 (14%)	32 (7%)	72 (15%)	------

[(a)] Geometry was optimized and the chemical partition was calculated with Sparkle/RM1.

Table S7. Spherical coordinates of all atoms belonging to the coordination polyhedron of the complexes of general formula $Eu(BTFA)_3(L)_2$ and $Eu(BTFA)_3(L,L')$, optimized with the RM1 model (except where otherwise indicated), where L and L' stand for the non-ionic ligands TPPO, DBSO, and PTSO.

Complex	*R* (Å)	θ (°)	φ (°)
$Eu(BTFA)_3(DBSO)_{2,Adj}$			
O (DBSO1)	2.3301	77.53	353.46
O (DBSO2)	2.3437	84.64	265.57
O (BTFA2)	2.3862	105.91	64.76
O (BTFA2)	2.4205	90.23	129.39
O (BTFA1)	2.4105	16.54	57.80
O (BTFA1)	2.3906	53.88	192.22
O (BTFA3)	2.4272	148.36	334.25
O (BTFA3)	2.3809	140.95	194.68
$Eu(BTFA)_3(DBSO)_{2,Non\text{-}Adj}$			
O (BTFA3)	2.4217	127.44	217.55
O (BTFA1)	2.3812	125.83	316.86
O (DBSO1)	2.3338	135.74	114.75
O (DBSO2)	2.3373	44.05	333.48
O (BTFA2)	2.3880	66.28	157.68
O (BTFA2)	2.4132	48.76	80.85
O (BTFA3)	2.3881	66.48	244.97
O (BTFA1)	2.4210	108.95	30.01
$Eu(BTFA)_3(DBSO,PTSO)_{Adj}$			
O (DBSO)	2.3410	90.36	359.18
O (PTSO)	2.3346	88.66	81.31
O (BTFA3)	2.4232	52.87	293.56
O (BTFA3)	2.3834	14.40	136.01
O (BTFA2)	2.3768	131.02	278.89
O (BTFA2)	2.4302	161.66	70.27
O (BTFA1)	2.4193	81.63	219.35
O (BTFA1)	2.3876	103.41	156.92
$Eu(BTFA)_3(DBSO,PTSO)_{Non\text{-}Adj}$			
O (PTSO)	2.3295	76.11	10.66
O (DBSO)	2.3399	84.45	171.48
O (BTFA3)	2.3926	153.27	35.78
O (BTFA3)	2.4144	106.91	103.42
O (BTFA1)	2.4261	30.47	93.01
O (BTFA1)	2.3793	36.36	257.16
O (BTFA2)	2.4237	101.98	302.37
O (BTFA2)	2.3892	126.39	233.49
$Eu(BTFA)_3(DBSO,TPPO)_{Adj}$			
O (TPPO)	2.3076	88.76	2.97

O (BTFA2)	2.4172	97.25	283.59
O (BTFA2)	2.4006	112.74	216.97
O (BTFA1)	2.4030	172.87	1.41
O (BTFA1)	2.4253	118.26	118.03
O (BTFA3)	2.4175	22.52	269.80
O (BTFA3)	2.4003	60.76	173.00
O (DBSO)	2.3586	58.64	84.18
$Eu(BTFA)_3(DBSO,TPPO)_{Non\text{-}Adj}$ [(a)]			
O (BTFA3)	2.4613	89.69	269.42
O (BTFA3)	2.4706	88.58	330.09
O (BTFA2)	2.4638	119.79	172.74
O (BTFA2)	2.4668	63.38	196.88
O (DBSO)	2.4191	166.05	301.50
O (TPPO)	2.3947	18.23	31.40
O (BTFA1)	2.4698	87.18	102.69
O (BTFA1)	2.4625	113.10	46.81
$Eu(BTFA)_3(PTSO)_{2,Adj}$			
O (PTSO1)	2.3435	49.65	150.47
O (BTFA1)	2.3897	62.88	2.28
O (BTFA1)	2.4263	74.26	72.28
O (BTFA2)	2.4163	116.09	214.97
O (BTFA2)	2.3879	126.11	137.04
O (BTFA3)	2.3904	143.55	28.94
O (BTFA3)	2.4257	117.57	302.96
O (PTSO2)	2.3338	55.95	265.75
$Eu(BTFA)_3(PTSO)_{2,Non\text{-}Adj}$			
O (BTFA2)	2.3983	89.32	266.68
O (BTFA2)	2.4271	88.58	332.83
O (PTSO1)	2.3352	109.29	196.13
O (BTFA3)	2.4167	126.42	93.05
O (BTFA3)	2.3970	161.43	329.15
O (PTSO2)	2.3324	65.12	39.97
O (BTFA1)	2.4111	20.31	237.07
O (BTFA1)	2.3895	60.10	134.77
$Eu(BTFA)_3(PTSO,TPPO)_{Adj}$			
O (BTFA1)	2.4253	90.19	121.00
O (BTFA1)	2.4030	85.10	55.77
O (BTFA3)	2.4045	109.96	276.47
O (BTFA3)	2.4339	67.07	327.30
O (BTFA2)	2.4074	133.50	3.92
O (BTFA2)	2.4098	159.76	161.47
O (TPPO)	2.3073	80.32	201.84
O (PTSO)	2.3586	8.47	52.05
$Eu(BTFA)_3(PTSO,TPPO)_{Non\text{-}Adj}$			

O (BTFA1)	2.4233	72.66	160.45
O (BTFA2)	2.4006	71.42	18.40
O (TPPO)	2.3471	145.99	8.39
O (BTFA3)	2.3966	108.52	228.92
O (BTFA3)	2.4298	95.95	295.05
O (PTSO)	2.3132	17.69	259.69
O (BTFA1)	2.4129	137.72	144.00
O (BTFA2)	2.4210	82.54	85.32
$Eu(BTFA)_3(TPPO)_{2,Adj}$			
O (BTFA1)	2.4332	89.84	320.08
O (TPPO1)	2.3137	86.10	243.61
O (TPPO2)	2.3193	5.95	39.95
O (BTFA2)	2.4338	134.11	176.14
O (BTFA2)	2.4221	74.54	145.86
O (BTFA3)	2.4396	115.63	84.67
O (BTFA3)	2.4000	159.62	351.69
O (BTFA1)	2.4284	86.59	25.78
$Eu(BTFA)_3(TPPO)_{2,Non\text{-}Adj}$			
O (BTFA1)	2.4395	91.77	142.08
O (TPPO1)	2.3212	92.79	39.98
O (BTFA2)	2.4032	88.55	322.62
O (TPPO2)	2.3159	86.57	219.38
O (BTFA1)	2.4028	35.85	104.00
O (BTFA3)	2.4028	151.52	107.85
O (BTFA2)	2.4383	29.53	289.81
O (BTFA3)	2.4390	144.28	281.99

[a] Geometry optimized with Sparkle/RM1.

Table S8. Spherical coordinates of all atoms belonging to the coordination polyhedron of the complexes of general formula $Eu(TTA)_3(L)_2$ and $Eu(TTA)_3(L,L')$ optimized with the RM1 model, where L and L' stand for the non-ionic ligands TPPO, DBSO, and PTSO.

Complex	*R* (Å)	θ (°)	φ (°)
$Eu(TTA)_3(DBSO)_{2,Adj}$			
O (DBSO1)	2.3336	76.85	352.05
O (TTA1)	2.4101	22.33	60.44
O (TTA1)	2.3880	52.27	183.25
O (TTA3)	2.4275	144.00	321.38
O (TTA2)	2.3861	112.84	60.98
O (TTA2)	2.4204	95.18	126.79
O (TTA3)	2.3749	139.46	198.72
O (DBSO2)	2.3396	77.12	262.76
$Eu(TTA)_3(DBSO)_{2,Non\text{-}Adj}$			
O (TTA1)	2.4224	94.65	98.44
O (TTA1)	2.3883	81.67	32.85
O (DBSO1)	2.3329	117.76	170.43
O (TTA2)	2.3872	157.88	22.22
O (TTA2)	2.4109	122.34	278.56
O (DBSO2)	2.3355	58.48	316.15
O (TTA3)	2.4226	64.66	221.76
O (TTA3)	2.3810	24.37	134.86
$Eu(TTA)_3(DBSO,PTSO)_{Adj}$			
O (PTSO)	2.3345	130.90	295.15
O (DBSO)	2.3373	96.63	204.08
O (TTA2)	2.4144	128.08	134.86
O (TTA2)	2.3896	130.7	44.86
O (TTA1)	2.3899	39.56	147.99
O (TTA1)	2.4241	67.53	69.45
O (TTA3)	2.4260	52.86	266.45
O (TTA3)	2.3755	61.29	346.76
$Eu(TTA)_3(DBSO,PTSO)_{Non\text{-}Adj}$			
O (DBSO)	2.3416	50.69	301.02
O (TTA1)	2.4258	30.75	114.74
O (PTSO)	2.3266	103.72	131.38
O (TTA3)	2.4227	142.72	51.76
O (TTA3)	2.3894	121.74	320.07
O (TTA2)	2.3874	132.09	219.09
O (TTA2)	2.4153	66.99	217.68
O (TTA1)	2.3807	68.81	36.99
$Eu(TTA)_3(DBSO,TPPO)_{Adj}$			
O (TTA3)	2.4035	86.53	115.79
O (TTA3)	2.4094	84.37	50.09
O (DBSO)	2.3571	110.86	255.40

O (TPPO)	2.3095	65.72	327.90
O (TTA2)	2.4220	147.01	358.90
O (TTA2)	2.3921	146.18	156.39
O (TTA1)	2.4004	17.21	144.77
O (TTA1)	2.4312	75.01	197.96
$Eu(TTA)_3(DBSO,TPPO)_{Non\text{-}Adj}$			
O (TTA2)	2.3894	90.38	269.66
O (TTA2)	2.4336	84.65	334.47
O (DBSO)	2.3470	166.12	295.02
O (TPPO)	2.3086	14.04	47.46
O (TTA3)	2.4188	64.29	203.37
O (TTA3)	2.3996	118.77	166.81
O (TTA1)	2.4290	89.00	103.35
O (TTA1)	2.3893	111.05	41.02
$Eu(TTA)_3(PTSO)_{2,Adj}$			
O (PTSO1)	2.3380	110.08	14.21
O (TTA3)	2.3870	149.35	258.06
O (TTA3)	2.4308	111.75	179.69
O (TTA2)	2.3847	63.46	241.95
O (TTA2)	2.4248	80.55	309.54
O (PTSO2)	2.3410	108.22	100.81
O (TTA1)	2.4169	37.44	31.94
O (TTA1)	2.3809	42.00	147.65
$Eu(TTA)_3(PTSO)_{2,Non\text{-}Adj}$			
O (PTSO1)	2.3355	78.65	289.86
O (PTSO2)	2.3335	80.24	104.88
O (TTA2)	2.3974	150.48	298.32
O (TTA2)	2.4256	141.70	141.69
O (TTA1)	2.3891	94.00	208.42
O (TTA1)	2.4204	30.46	189.44
O (TTA3)	2.4144	108.73	25.70
O (TTA3)	2.3855	43.87	14.82
$Eu(TTA)_3(PTSO,TPPO)_{Adj}$			
O (TPPO)	2.3071	90.89	357.18
O (TTA1)	2.4232	82.43	275.07
O (TTA1)	2.4026	64.99	208.97
O (TTA2)	2.4002	123.99	175.9
O (TTA2)	2.4144	157.08	283.40
O (PTSO)	2.3596	9.31	55.56
O (TTA3)	2.4336	71.52	117.61
O (TTA3)	2.3986	123.89	76.69
$Eu(TTA)_3(PTSO,TPPO)_{Non\text{-}Adj}$			
O (PTSO)	2.3404	37.03	47.61
O (TTA1)	2.4293	110.26	335.04

O (TPPO)	2.3094	145.83	233.71
O (TTA2)	2.3980	60.34	147.79
O (TTA2)	2.4244	119.09	120.02
O (TTA3)	2.4336	68.58	221.51
O (TTA3)	2.3939	55.04	294.43
O (TTA1)	2.3955	112.24	45.00
$Eu(TTA)_3(TPPO)_{2,Adj}$			
O (TTA2)	2.4318	88.26	265.57
O (TTA2)	2.4229	91.77	331.05
O (TTA3)	2.4396	20.64	309.98
O (TTA3)	2.3983	59.53	203.57
O (TPPO1)	2.3196	110.29	47.11
O (TTA1)	2.4314	99.76	140.88
O (TTA1)	2.4221	45.96	99.42
O (TPPO2)	2.3143	158.55	218.68
$Eu(TTA)_3(TPPO)_{2,Non\text{-}Adj}$			
O (TTA1)	2.4304	89.95	321.81
O (TPPO1)	2.3212	161.68	80.36
O (TTA2)	2.3969	90.82	181.12
O (TTA2)	2.4473	86.59	115.67
O (TPPO2)	2.3248	17.05	64.53
O (TTA3)	2.4343	124.13	255.02
O (TTA3)	2.4073	58.32	251.14
O (TTA1)	2.4084	91.99	27.21

Table S9. Spherical coordinates of all atoms belonging to the coordination polyhedron of the complexes of general formula $Eu(DBM)_3(L)_2$ and $Eu(DBM)_3(L,L')$ optimized with the RM1 model (except where otherwise indicated), where β-diketonate stands for the ionic ligands DBM; and L and L' stand for the non-ionic ligands TPPO, DBSO, and PTSO.

Complex	*R* (Å)	θ (°)	φ (°)
$Eu(DBM)_3(DBSO)_{2,Adj}$			
O (DBSO1)	2.3475	80.22	196.07
O (DBSO2)	2.3634	92.76	117.95
O (DBM1)	2.3985	89.34	265.97
O (DBM1)	2.3833	89.76	332.23
O (DBM2)	2.3899	8.62	250.62
O (DBM2)	2.3852	57.64	48.97
O (DBM3)	2.3882	160.01	215.12
O (DBM3)	2.3861	134.15	44.34
$Eu(DBM)_3(DBSO)_{2,Non\text{-}Adj}$			
O (DBM1)	2.3909	9.19	109.11
O (DBM1)	2.3822	72.81	81.95
O (DBM2)	2.3933	81.81	267.21
O (DBM2)	2.3958	72.51	333.58
O (DBSO1)	2.3485	73.06	196.96
O (DBM3)	2.3860	123.81	138.45
O (DBM3)	2.3825	159.47	249.40
O (DBSO2)	2.3472	120.22	25.46
$Eu(DBM)_3(DBSO,PTSO)_{Adj}$			
O (DBM1)	2.3896	94.92	253.98
O (DBM1)	2.3882	61.22	313.48
O (DBM2)	2.4016	81.15	96.71
O (DBM2)	2.3780	84.25	30.15
O (PTSO)	2.3616	14.83	166.32
O (DBSO)	2.3421	95.23	176.76
O (DBM3)	2.3925	155.98	121.48
O (DBM3)	2.3861	136.96	326.35
$Eu(DBM)_3(DBSO,PTSO)_{Non\text{-}Adj}$			
O (DBSO)	2.3541	129.75	70.70
O (DBM1)	2.3942	101.52	292.86
O (DBM1)	2.3927	98.88	358.81
O (DBM2)	2.3826	88.33	204.10
O (DBM2)	2.3930	154.00	212.43
O (PTSO)	2.3426	28.31	265.67
O (DBM3)	2.3956	46.90	62.57
O (DBM3)	2.3833	76.90	131.17
$Eu(DBM)_3(DBSO,TPPO)_{Adj}$			
O (DBSO)	2.3859	106.81	295.92

O (TPPO)	2.3164	56.43	225.63
O (DBM1)	2.4013	103.65	14.93
O (DBM2)	2.3980	117.97	172.79
O (DBM2)	2.4001	174.09	294.36
O (DBM3)	2.4001	107.99	89.96
O (DBM3)	2.3815	46.62	114.09
O (DBM1)	2.3927	41.26	354.21
$Eu(DBM)_3(DBSO,TPPO)_{Non-Adj}$			
O (DBSO)	2.3554	149.65	7.44
O (TPPO)	2.3236	9.13	173.01
O (DBM1)	2.3942	88.97	119.02
O (DBM1)	2.3927	86.99	54.22
O (DBM2)	2.3891	99.91	280.59
O (DBM2)	2.4046	71.65	340.02
O (DBM3)	2.4056	143.96	178.49
O (DBM3)	2.3917	83.73	208.19
$Eu(DBM)_3(PTSO)_{2,Adj}$			
O (DBM1)	2.3821	136.36	55.58
O (PTSO1)	2.3625	57.47	180.15
O (PTSO2)	2.3470	134.52	193.34
O (DBM2)	2.3822	69.75	272.20
O (DBM2)	2.3908	128.00	304.40
O (DBM3)	2.3890	76.90	1.35
O (DBM3)	2.4009	21.61	57.94
O (DBM1)	2.4022	87.23	104.99
$Eu(DBM)_3(PTSO)_{2,Non-Adj}$			
O (DBM1)	2.3924	8.32	285.22
O (DBM1)	2.3891	56.67	91.68
O (DBM2)	2.3878	95.93	263.61
O (DBM2)	2.3968	77.93	327.40
O (PTSO1)	2.3467	77.32	189.95
O (DBM3)	2.3927	123.68	129.36
O (DBM3)	2.3905	168.71	270.40
O (PTSO2)	2.3517	107.03	33.68
$Eu(DBM)3(PTSO,TPPO)_{Adj}$			
O (PTSO)	2.3837	65.56	20.35
O (TPPO)	2.3168	35.29	139.55
O (DBM1)	2.4036	142.02	21.72
O (DBM3)	2.4027	60.79	250.46
O (DBM3)	2.4039	86.46	314.38
O (DBM2)	2.4024	134.81	252.03
O (DBM2)	2.3841	112.63	174.96
O (DBM1)	2.3985	109.08	95.78
$Eu(DBM)_3(PTSO,TPPO)_{Non-Adj}$			

O (DBM1)	2.4059	86.52	103.88
O (PTSO)	2.3626	18.70	52.54
O (DBM2)	2.3995	85.01	249.52
O (DBM2)	2.3945	62.05	185.48
O (TPPO)	2.3232	147.11	169.92
O (DBM3)	2.4118	66.11	322.38
O (DBM1)	2.3867	103.88	40.02
O (DBM3)	2.3939	132.65	320.51
$Eu(DBM)_3(TPPO)_{2,Adj}$ [(a)]			
O (DBM1)	2.4226	95.30	56.56
O (TPPO1)	2.3285	101.61	199.25
O (DBM2)	2.3284	164.95	351.98
O (TPPO2)	2.4324	47.92	256.94
O (DBM2)	2.3938	97.98	302.37
O (DBM3)	2.4075	54.64	2.95
O (DBM3)	2.3938	24.45	115.37
O (DBM1)	2.4046	98.21	122.51
$Eu(DBM)_3(TPPO)_{2,Non\text{-}Adj}$			
O (TPPO1)	2.4241	91.31	48.07
O (TPPO2)	2.4240	92.74	201.20
O (DBM1)	2.4637	46.00	128.14
O (DBM3)	2.4657	77.03	274.19
O (DBM2)	2.4621	172.45	276.58
O (DBM1)	2.4637	20.27	346.01
O (DBM2)	2.4686	123.79	124.72
O (DBM3)	2.4671	105.73	331.65

[(a)] Geometry optimized with Sparkle/PM3.

Reference

1. Dutra, J.D.L., Lima, N.B.D., Freire, R.O. & Simas, A.M. Europium Luminescence: Electronic Densities and Superdelocalizabilities for a Unique Adjustment of Theoretical Intensity Parameters. *Sci. Rep.* **5**, 13695 (2015).
